# The Comparability of English, French and Dutch Scores on the Functional Assessment of Chronic Illness Therapy-Fatigue (FACIT-F): An Assessment of Differential Item Functioning in Patients with Systemic Sclerosis

**DOI:** 10.1371/journal.pone.0091979

**Published:** 2014-03-17

**Authors:** Linda Kwakkenbos, Linda M. Willems, Murray Baron, Marie Hudson, David Cella, Cornelia H. M. van den Ende, Brett D. Thombs, and the Canadian Scleroderma Research Group

**Affiliations:** 1 Department of Psychiatry, McGill University, Montréal, Québec, Canada; 2 Lady Davis Institute for Medical Research, Jewish General Hospital, Montréal, Québec, Canada; 3 Department of Rheumatology, Sint Maartenskliniek Nijmegen, The Netherlands; 4 Department of Medicine, McGill University, Montréal, Québec, Canada; 5 Department of Medical Social Sciences, Northwestern University Feinberg School of Medicine, Chicago, Illinois, United States of America; 6 Departments of Epidemiology, Biostatistics, and Occupational Health, McGill University, Montréal, Québec, Canada; 7 Educational and Counselling Psychology, McGill University, Montréal, Québec, Canada; 8 Psychology, McGill University, Montréal, Québec, Canada; 9 School of Nursing, McGill University, Montréal, Québec, Canada; University of Texas Health Science Center at Houston, United States of America

## Abstract

**Objective:**

The Functional Assessment of Chronic Illness Therapy- Fatigue (FACIT-F) is commonly used to assess fatigue in rheumatic diseases, and has shown to discriminate better across levels of the fatigue spectrum than other commonly used measures. The aim of this study was to assess the cross-language measurement equivalence of the English, French, and Dutch versions of the FACIT-F in systemic sclerosis (SSc) patients.

**Methods:**

The FACIT-F was completed by 871 English-speaking Canadian, 238 French-speaking Canadian and 230 Dutch SSc patients. Confirmatory factor analysis was used to assess the factor structure in the three samples. The Multiple-Indicator Multiple-Cause (MIMIC) model was utilized to assess differential item functioning (DIF), comparing English versus French and versus Dutch patient responses separately.

**Results:**

A unidimensional factor model showed good fit in all samples. Comparing French versus English patients, statistically significant, but small-magnitude DIF was found for 3 of 13 items. French patients had 0.04 of a standard deviation (SD) lower latent fatigue scores than English patients and there was an increase of only 0.03 SD after accounting for DIF. For the Dutch versus English comparison, 4 items showed small, but statistically significant, DIF. Dutch patients had 0.20 SD lower latent fatigue scores than English patients. After correcting for DIF, there was a reduction of 0.16 SD in this difference.

**Conclusions:**

There was statistically significant DIF in several items, but the overall effect on fatigue scores was minimal. English, French and Dutch versions of the FACIT-F can be reasonably treated as having equivalent scoring metrics.

## Introduction

Chronic fatigue from medical illness can be characterized as persistent exhaustion that is disproportionate to exertion and not relieved by rest. Fatigue is common and often persistent in rheumatic diseases and can have a major impact on health-related quality of life (HRQL)[1], [2]. Patients with systemic sclerosis (SSc, or scleroderma), a chronic, multi-system connective tissue disorder characterized by thickening and fibrosis of the skin, involvement of internal organs, substantially reduced HRQL, and significant morbidity and mortality [3]–[5] report that fatigue impacts HRQL as much or more than any other symptom [6]–[8]. Fatigue was reported to be present in 89% of 464 Canadian SSc patients who responded to a national survey, with an impact on the ability to carry out daily activities in 72% [9]. A Dutch study found that 92% of 123 patients were bothered by fatigue [8]. Fatigue in SSc is independently associated with reduced capacity to carry out daily activities, work disability and impaired physical function [10]–[13]. Fatigue ratings by SSc patients are similar to those of patients with other rheumatic diseases and cancer patients currently undergoing treatment, and substantially worse than in the general population or among cancer patients in remission [14].

Several instruments have been used to assess fatigue in rheumatic diseases [15],[16]. Compared to other measures, the Functional Assessment of Chronic Illness Therapy- Fatigue (FACIT-F) has been found to provide better coverage of the full range of the fatigue spectrum in SSc [17] and rheumatoid arthritis [18]. This is important because SSc patients are in the moderate to severe range of fatigue, but the SF-36 vitality subscale, for instance, targets the healthy end of the spectrum and does not differentiate between patients with moderate versus severe fatigue [17], [18]. The Multidimensional Assessment of Fatigue (MAF) scale, on the other hand, best discriminates between patients in the middle of the spectrum, but does not differentiate well between patients with moderately high versus severe fatigue or moderately low versus very low fatigue [18].

The FACIT-F has been translated into more than 50 languages, which is important when outcomes are reported in multiple languages, including in countries with more than one common language, such as Canada (French/English) or the United States (Spanish/English), as well as in international multi-center collaborations, which are utilized frequently in rare diseases, such as SSc. However, to pool results from the FACIT-F among study participants from different countries or to compare results between patients from different cultural or linguistic groups, it is necessary to establish *measurement equivalence*, meaning that patients across language groups with similar levels of fatigue will have similar scores on FACIT-F items [19]. Differential item functioning (DIF) is said to occur when patients from different cultural or linguistic groups with similar levels of a construct, such as fatigue, score differently on an item assessing fatigue. DIF in cross-linguistic comparisons may occur because translations shift meanings, formats, or severity of items used in patient-reported outcome measures, which can lead to responses that differ across groups even when levels of the outcome being measured are similar [20].

The objective of the present study was to assess the cross-language measurement equivalence of the English, French, and Dutch versions of the FACIT-F scale in SSc patients.

## Methods

### Ethics Statement

The English-speaking and French-speaking samples of this study consisted of patients with SSc enrolled in the Canadian Scleroderma Research Group Registry (CSRG). The study was approved by the Institutional Review Board of McGill University and all patients provided written consent for their information to be stored in a computer database and used for research. The Dutch sample consisted of members of the Dutch organization for patients with systemic autoimmune diseases (NVLE). The organization mailed members with SSc an invitation to complete the online survey or a paper version on request. Ethical approval was obtained from the Institutional Review Board of the Radboud University Medical Center Nijmegen. According to Dutch regulations, signed informed consent was not required because of the non-invasive nature of the study.

### Patients and Procedures

#### English- and French-speaking samples

The English and French-speaking samples consisted of patients who completed the FACIT-F from November 2007 through March 2013 in the Canadian Scleroderma Research Group (CSRG) Registry. Patients with a diagnosis of SSc confirmed by a CSRG rheumatologist, who are at least18 years of age and fluent in English or French are recruited for the Registry from 15 centers across Canada. Patients in the Registry undergo extensive physical evaluations at annual visits and complete a series of self-report questionnaires in their preferred language (English or French). For patients who completed the FACIT-F at multiple annual visits, the first available visit with complete FACIT-F data was used.

*Dutch sample.* The Dutch sample consisted of members of the Dutch patient organization for patients with systemic autoimmune diseases (NVLE). The NVLE mailed members with SSc an invitation to complete an anonymous online survey, or a paper version on request, between June and August 2011. The survey consisted of a series of self-report questionnaires related to fatigue, health care utilization, and HRQL. Patients with a self-reported diagnosis of limited or diffuse SSc who were 18 years of age or older were included in this study.

### Measures

#### Demographics and disease characteristics

Demographic variables available in all three samples included age, sex, marital status, education, current employment status, time since diagnosis, and SSc subtype. In the English and French samples, time since diagnosis and a patient's classification as having limited or diffuse SSc were provided by a CSRG rheumatologist. Limited SSc was defined as skin involvement distal to the elbows and knees only, whereas diffuse SSc was defined as skin involvement proximal to the elbows and knees, and/or the trunk [21]. In the Dutch sample, both time since diagnosis and SSc subtype were patient-reported.

#### Functional Assessment of Chronic Illness Therapy- Fatigue (FACIT-F)

The FACIT-F consists of 13 items that assesses tiredness, weakness and difficulty conducting everyday activities due to fatigue in the past 7 days [22]. Items are scored on a 5-point scale (0  =  not at all, 4  =  very much). All items except items 7 (*I have energy*) and 8 (*I am able to do my usual activities*) are reverse-scored before item scores are summed to obtain a total score (range 0–52). Higher scores reflect less fatigue. The FACIT has been shown to have excellent internal consistency (Cronbach's alpha >0.90) and very good concurrent, divergent and predictive validity across several patient populations [18]. The original English, French and Dutch versions of the FACIT-F were used [23].

### Statistical Analyses

For all comparisons, the English-speaking sample was used as the reference group. Demographics and disease characteristics were compared between the English and French samples, and between the English and Dutch samples using the chi-square statistic for categorical variables and t-tests for continuous variables.

The factor structure of the FACIT-F was assessed for each sample separately using confirmatory factor analysis (CFA). Ideally for DIF assessment, the simplest structure with reasonable fit is used. The FACIT-F has shown to have a single-dimensional factor structure across diverse samples [24]. Thus, a single-dimensional CFA model was constructed to determine whether this structure could be reasonably used in the DIF analysis. Item responses for the FACIT-F were ordinal Likert data and were therefore modeled using the weighted least squares estimator with a diagonal weight matrix, robust standard errors, and a mean- and variance-adjusted chi-square statistic with delta parameterization [25]. The chi-square test, the Tucker-Lewis Index (TLI) [26], the Comparative Fit Index (CFI) [27] and the Root Mean Square Error of Approximation (RMSEA) [28] were used to assess model fit. Good fitting models are indicated by a TLI and CFI ≥0.95 and RMSEA ≤0.06 [29], although a CFI of .90 or above [30] and a RMSEA of .08 or less [31] are often regarded as indicators of acceptable model fit. Since the chi-square test is highly sensitive to sample size, it can lead to the rejection of well-fitting models [32]. Therefore, the TLI, CFI and RMSEA fit indices were emphasized. Modification indices were used to identify pairs of items for which model fit would improve if error estimates were freed to covary and for which there appears to be theoretically justifiable shared method effects (e.g., similar wording) [33]. Once the factor structure was established for each sample separately, a CFA model was fit that included patients from English and French samples and English and Dutch samples combined, respectively.

To determine if items of the FACIT-F exhibited DIF for French versus English and Dutch versus English, the Multiple-Indicator Multiple-Cause (MIMIC) model was utilized. MIMIC models for DIF assessment are based on structural equation models, in which the grouping variable (language) is added to the basic CFA model as an observed variable. The base MIMIC model consists of the CFA factor model, to which the additional direct effect of group on the latent factors is added. This serves to control for group differences on the level of the latent factors. An important strength of the MIMIC model is that it allows for adjustment for important covariates that may differ between comparison groups, by adding a direct effect of these variables on the latent factors. We controlled for differences between samples in age, sex, marital status, education, current employment status, SSc subtype, and disease duration.

Each FACIT-F item was regressed separately on the language variable to assess potential DIF. Statistically significant DIF is represented by a statistically significant association in the model from language to the item, while controlling for any differences in the overall level of the latent factor between groups (by regressing the latent factor on language). If there was DIF for one or more items, the item with the largest magnitude of DIF was considered to have DIF, and the association between the linguistic group variable and that item was included in the model. This procedure was repeated until none of the remaining items show significant DIF. Once all items with significant DIF were identified, the potential magnitude of DIF items collectively was evaluated by comparing the difference on the latent factor between groups in the baseline CFA model and after controlling for DIF. The magnitude of this difference was interpreted following Cohen's effect sizes, with ≤0.20 SD indicating small, 0.50 SD  =  moderate and 0.80 SD  =  large differences [34], [35], [36].

For the English versus French and English versus Dutch comparisons, separately, Hommels' correction for multiple testing was applied [37]. CFA and DIF analyses were conducted using Mplus 7 [25] and all other analyses were conducted using IBM SPSS Statistics 20 (Chicago, IL).

## Results

### Sample characteristics

Demographic and disease characteristics for the three samples are displayed in Table 1.

**Table 1:** Demographic and disease characteristics for the three SSc samples.

Variable	English (N = 871)	French (N = 238)	Dutch (N = 230)
Female (%)	755 (86.7)	211 (88.7)	193 (83.9)
Mean age, years (SD)	56.6 (12.1)	57.8 (10.4)	58.3 (11.1)^a^
Higher education (%>12 years)	433 (49.8)^b^	101 (42.4)*	106 (46.5)^a^
Currently working (%)	355 (40.8)^c^	91 (38.2)	48 (21.1)^a^ **
Married or living as married (%)	728 (83.6)	188 (79.0)	165 (71.7) **
Limited disease (%)	568 (69.1)^d^	149 (63.9)	147 (66.5)^e^
Time since diagnosis, mean (SD)	9.2 (8.4)^f^	8.2 (8.6)	11.0 (9.3)^g^*
FACIT-F score, mean (SD)	32.5 (12.1)	31.5 (12.2)	29.1 (10.4)**

Due to missing values: ^a^N = 228, ^b^N = 869,^ c^N = 868, ^d^N = 822, ^e^N = 221, ^f^N = 861, ^g^N = 225.

Difference with English sample: *P<0.05; **P<0.001.

#### English sample

The English sample consisted of 871 patients who completed the FACIT-F, with a mean age of 56.6 years (SD = 12.1) and mean time since diagnosis of 9.2 years (SD = 8.4). The majority (86.7%) were female and most patients were married or living as married (83.6%). The mean FACIT-F score was 32.5 (SD = 12.1).

#### French sample

In **t**otal, 238 patients completed the FACIT-F in French. The mean age was 57.8 years (SD = 10.4) and the mean time since diagnosis was 8.2 years (SD = 8.6). The majority (88.7%) were female and had a partner (79.0%). The mean FACIT-F score was 31.5 (SD = 12.2). Patients in the French sample were less likely to have >12 years of education than patients in the English sample (P<0.05).

#### Dutch sample

A total of 230 patients completed the FACIT-F in Dutch. The mean age was 58.3 years (SD = 11.1) and mean time since diagnosis was 11.0 years (SD = 9.3). Most patients were female (83.9%) and married or living as married (71.7%). The mean FACIT-F score was 29.1 (SD = 10.4). Dutch patients were less likely to be currently working or to be married than patients in the English sample. Furthermore, patients in the Dutch sample had significantly longer time since diagnosis and lower (worse) mean FACIT-F scores than the English sample (P<0.05).

### Confirmatory factor analysis

A single-factor structure was initially assessed in all three samples separately (English: Χ^2^(65) = 1416.5, P<0.001, CFI = 0.97, TLI = 0.97, RMSEA = 0.16; French: Χ^2^(65) = 325.2, P<0.001, CFI = 0.98, TLI = 0.98, RMSEA = 0.13; Dutch: Χ^2^(65) = 345.6, P<0.001, CFI = 0.97, TLI = 0.96, RMSEA = 0.14). Inspection of the modification indices indicated that freeing error terms to covary for items 5 (‘trouble starting things’) and 6 (‘trouble finishing things’), items 7 (‘energy’) and 8 (‘ability to do usual activities’), and items 1 (‘fatigued’) and 4 (‘tired’) would improve model fit, and there was clearly recognizable overlap in the item's content for items 5 and 6, as well as 1 and 4. Items 7 and 8 are the two only reverse-scored items of the FACIT-F and may therefore have more shared method effects compared to other items. This change resulted in a model with good enough fit in all three samples to be treated as a unidimensional construct for the purpose of DIF assessment (English: Χ^2^(62) = 873.3, P<0.001, CFI = 0.98, TLI = 0.98, RMSEA = 0.12; French: Χ^2^(62) = 193.5, P<0.001, CFI = 0.99, TLI = 0.99, RMSEA = 0.09; Dutch: Χ^2^(62) = 152.81, P<0.001, CFI = 0.99, TLI = 0.99, RMSEA = 0.08).

### Differential Item Functioning

#### French versus English

The single-factor structure was fit to the combined English and French sample, including a direct effect of language (English/French) on the latent fatigue factor and direct effects of covariates on the latent fatigue factor, to correct for differences in latent fatigue levels between the samples and differences in sample characteristics, respectively. The single-factor model showed good fit (Χ^2^(158) = 1197.6, P<0.001, CFI = 0.98, TLI = 0.98, RMSEA = 0.08). Prior to accounting for possible DIF, French patients had 0.04 SD lower latent factor scores (more fatigue) than English patients, although this difference was not statistically significant (95% confidence interval [CI] -0.15 to 0.11, P = 0.63) Three items showed statistically significant DIF: item 1 (z = 9.34, P<0.001), item 4 (z = 4.46, P<0.001), and item 8 (z = 7.38, P<0.001). Items 1 and 8 had higher scores (less fatigue) in the French sample compared with the English sample, while item 4 had lower scores in the French sample compared with the English sample (Table 2).

**Table 2:** Factor loadings for the FACIT-F in English and French samples and influence on the overall estimates of fatigue latent factor scores.

	Base model^a^		DIF corrected model^b^	
	Factor loading	95% Confidence Interval	Factor loading	95% Confidence Interval
**FACIT-F items:**				
1. I feel fatigued	0.87	[0.85, 0.89]	0.87	[0.85, 0.89]
2. I feel weak all over	0.85	[0.83, 0.87]	0.85	[0.83, 0.87]
3. I feel listless (“washed out”)	0.90	[0.89, 0.92]	0.90	[0.89, 0.92]
4. I feel tired	0.91	[0.89, 0.92]	0.91	[0.89, 0.92]
5. I have trouble starting things because I am tired	0.92	[0.90, 0.93]	0.92	[0.90, 0.93]
6. I have trouble finishing things because I am tired	0.90	[0.88, 0.91]	0.90	[0.88, 0.91]
7. I have energy	0.69	[0.66, 0.72]	0.69	[0.66, 0.72]
8. I am able to do my usual activities	0.59	[0.55, 0.63]	0.59	[0.55, 0.63]
9. I need to sleep during the day	0.66	[0.62, 0.70]	0.66	[0.62, 0.70]
10. I am too tired to eat	0.71	[0.66, 0.75]	0.71	[0.66, 0.75]
11. I need help doing my usual activities	0.71	[0.67, 0.74]	0.71	[0.67, 0.74]
12. I am frustrated by being too tired to do the things I want to do	0.89	[0.87, 0.90]	0.89	[0.87, 0.90]
13. I have to limit my social activity because I am tired	0.88	[0.86, 0.90]	0.88	[0.86, 0.90]
**Direct effects on items attributable to French language:**				
**Item 1**			0.42	[0.33, 0.52]
**Item 4**			−0.47	[−0.60, −0.34]
**Item 8**			0.20	[0.11, 0.28]
**Structural effect of English language of latent factors**	−0.04	[−0.19, 0.11]	−0.07	[−0.22, 0.08]

a Not corrected for DIF, ^b^Corrected for DIF for item 1, 4 and 8.

As shown in Table 2, after correcting for DIF, compared with the base model, there was an increase of only 0.03 SD on the latent fatigue factor in the difference between English and French samples, for a between-groups difference of 0.07 (95% CI −0.22 to 0.08, P = 0.79). Thus, although there was statistically significant DIF on 3 items, this did not influence the overall latent factor scores of French versus English scores substantially.

#### Dutch versus English

The single-factor structure was fit to the combined English and Dutch sample, along with a direct effect of language (English/Dutch) and the covariates on the latent factor, showing good fit (Χ^2^(158) = 1107.5, P<0.001, CFI = 0.98, TLI = 0.98, RMSEA = 0.08). Prior to accounting for possible DIF, Dutch patients had 0.20 SD lower latent factor scores (more fatigue) than English patients, and this difference was statistically significant (95% CI −0.36 to −0.04, P = 0.01). Four items showed statistically significant DIF: item 7 (z = 10.0, P<0.001), item 8 (z = 6.40, P<0.001), item 9 (z = 3.51, P<0.001), and item 13 (z = 3.81, P<0.001). All four items had lower scores (more fatigue) in the Dutch sample compared with the English sample.

After correcting for DIF, compared with the base model, there was a reduction of 0.16 SD in the difference between English and Dutch samples as shown in Table 3, and between-group differences were no longer significant (−0.04 SD, 95% CI −0.21 to 0.08, P = 0.17). The magnitude of the difference, however, in overall fatigue was small, even though 4 items had statistically significant DIF.

**Table 3:** Factor loadings for the FACIT-F in English and Dutch samples and influence on the overall estimates of fatigue latent factor scores.

	Base model^a^		DIF corrected model^b^	
	Factor loading	95% Confidence Interval	Factor loading	95% Confidence Interval
**FACIT-F items:**				
1. I feel fatigued	0.86	[0.84, 0.88]	0.86	[0.84, 0.88]
2. I feel weak all over	0.84	[0.82, 0.86]	0.84	[0.82, 0.86]
3. I feel listless (“washed out”)	0.88	[0.87, 0.90]	0.88	[0.87, 0.90]
4. I feel tired	0.90	[0.88, 0.92]	0.90	[0.89, 0.92]
5. I have trouble starting things because I am tired	0.91	[0.90, 0.93]	0.91	[0.90, 0.93]
6. I have trouble finishing things because I am tired	0.88	[0.87, 0.90]	0.88	[0.87, 0.90]
7. I have energy	0.68	[0.64, 0.71]	0.68	[0.64, 0.71]
8.I am able to do my usual activities	0.55	[0.51, 0.59]	0.55	[0.51, 0.59]
9. I need to sleep during the day	0.63	[0.58, 0.67]	0.63	[0.58, 0.67]
10. I am too tired to eat	0.68	[0.63, 0.72]	0.68	[0.63, 0.72]
11. I need help doing my usual activities	0.69	[0.65, 0.72]	0.69	[0.65, 0.72]
12.I am frustrated by being too tired to do the things I want to do	0.87	[0.85, 0.89]	0.87	[0.85, 0.89]
13.I have to limit my social activity because I am tired	0.86	[0.84, 0.88]	0.86	[0.84, 0.88]
**Direct effects on items attributable to Dutch language:**				
**Item 7**			−0.74	[−0.87, −0.64]
**Item 8**			−0.57	[−0.73, −0.40]
**Item 9**			−0.28	[−0.42, −0.13]
**Item 13**			−0.24	[−0.36, −0.04]
**Structural effect of English language on latent factors:**	−0.20	[−0.36, −0.04]	−0.04	[−0.21, 0.13]

a Not corrected for DIF, ^b^Corrected for DIF for item 7, 8, 9, and 13.

As a sensitivity analysis, we ran the MIMIC model with the 9 items that had no statistically significant DIF, yielding virtually the same results as the 13-item model corrected for the 4 DIF items, with a factor loading for language on the latent factor of −0.04.

## Discussion

The main finding of this study was that, although there were some items with statistically significant DIF, the magnitude of the DIF was small, and there were not substantive differences in measurement between French and English, and Dutch and English version of the FACIT-F. There was statistically significant DIF for 3 of 13 items in French and 4 items in Dutch compared with the original English version. French patients had higher FACIT-F scores (less fatigue) on items 1 and 8, and lower scores on item 4. Dutch patients had lower scores (more fatigue) on items 7, 8, 9, and 13 compared to the English sample. The influence of DIF on the overall fatigue estimates, however, was negligible for the French-English comparison. For the Dutch translation, the influence of DIF on latent fatigue factor levels was larger, but still small (i.e., ≤0.20 SD), suggesting that FACIT-F scores from English- and Dutch-speaking samples can also be validly compared and assumed to measuring fatigue using substantively the same metric.

Where there is differential item functioning, it may be related to translational differences. For the French items that were identified with DIF, only item 1 appeared to have a potentially meaningful difference from the English version. In item 1, the English ‘fatigued’ is translated as the French ‘épuisée’, which may be interpreted as ‘exhausted’. Exhaustion, however, is generally considered a more severe case of fatigue [38], which may have influenced the higher (reflecting less fatigue) scores of French SSc patients for this item.

In the English-Dutch comparison, the amount of DIF was largest for items 7 and 8. For item 7 (*I have energy*), the Dutch translation might be best understood as ‘I feel energetic’ (*Ik voel me energiek*). Feeling energetic, however, may be suggestive of having a high amount of energy, and people who have energy may not necessarily feel energetic. This distinction may have played a role in the lower fatigue scores (worse) on this item in the Dutch sample.

It has been previously noted that FACIT-F item 8 (*I am able to do my usual activities*) could be misinterpreted as a measure of fatigue in rheumatic diseases [16]. Because the item includes no direct reference to fatigue, ‘ability’ could be interpreted as a consequence of, for instance, physical limitations due to SSc, rather than fatigue. Item 8 was found to have a very low factor loading in our Dutch sample (0.35), which was much lower than any other factor loadings (0.56 to 0.90). This was not the case, however, for the English and French models, where the factor loading for item 8 in the English (0.61) and French (0.61) samples was similar to the range of factor loadings for other items (English, 0.66 to 0.92; French 0.65 to 0.96). It is not known why this item was differentially associated with fatigue in the Dutch sample, but, again, translation may be a factor. The Dutch word (‘gewone’) that was chosen to translate ‘usual’ is more closely related to the English ‘normal’. Normal activities, however, may suggest activities done by people not confronted with a disease, such as SSc, whereas ‘usual’ in English, may be interpreted as ‘everyday activities.’

Despite these item differences, overall, there was no evidence that the DIF items for the Dutch translation influenced fatigue scores in any more than a trivial magnitude. Therefore, scores generated with the FACIT-F in English, French, and Dutch SSc patients can be reasonably treated as comparable without adjustment for linguistic differences. Nonetheless, if our findings are replicated, the translations of some items, particularly the Dutch translations of items 7 and 8, might be reconsidered, especially given the influence of the FACIT system in other approaches to measure fatigue in chronic diseases, including the development of different item banks for Computer Adaptive Testing [39]–[41].

Effective research often requires international collaboration to include a sufficient number of patients for adequately powered studies, particularly in rare diseases. In SSc, for instance, the Scleroderma Clinical Trials Consortium [42] and the EULAR Scleroderma Trials and Research group [43] routinely conduct multicenter drug trials involving patients who complete outcome measures in multiple different languages. In addition, the Scleroderma Patient-centered Intervention Network (SPIN) was recently organized to test psychosocial and rehabilitation interventions in patients from across Canada, the US, and Europe [44], [45]. Improvement of fatigue management will be an important target for SPIN interventions. The current study supports the use of the FACIT-F in the different languages included in SPIN, and future studies should extend this assessment of the FACIT-F into other languages. In addition, measurement equivalence should also be assessed for other frequently used patient-reported outcome measures central to research in rheumatic diseases.

There are limitations that should be considered in interpreting the results of this study. Because of the difference in sample size between the samples, the core model used to assess DIF relied more on data from English-speaking patients than French and Dutch patients. However, since the initial factor analysis yielded the same results in all three samples, it does not seem likely that this would have influenced results substantially. It should be noted that in all three samples, the RMSEA exceeded the commonly used 0.06 threshold. This is similar to what has been found in other samples in which the factor structure of the FACIT-F was assessed [24]. The excellent CFI and TLI parameters in our samples, on the other hand, suggest the essential unidimensionality of the FACIT-F. In addition, when improving model fit by identifying pairs of items for which error estimates were freed to covary, there is no objective standard to assess whether there are theoretically justifiable shared method effects, such as similar wording. Other limitations relate to differences in sample recruitment between the Dutch and Canadian English and French samples. Whereas the English-speaking patients were recruited from 15 centers from across Canada, Dutch patients were recruited through the Dutch patient organization. Therefore, medical data in the English and French samples were based on medical records, in contrast to the Dutch sample for which these were self-reported, and there were large differences in disease duration. However, the analysis correcting for differences in demographics and disease characteristics between samples yielded virtually the same results as the non-corrected model, which suggests that differences in sampling did not likely influence the results substantially. In addition, our English-speaking and French-speaking data were both collected from Canadian patients. Both language and cultural differences related to the construct being measured may affect measurement, and thus, DIF. Therefore, it remains to be elucidated to which extend our results generalize to other French-speaking countries. Finally, a potential disadvantage of the MIMIC model, that was used in the present study, compared with other models to assess DIF is, that MIMIC does not test for non-uniform DIF. Non-uniform DIF means that the amount of DIF is unequal for different levels of the outcome of interest, in our case fatigue. On the other hand, MIMIC models do allow for adjustment for important covariates that may differ between comparison groups, which is an important strength of the model, especially given the differences in sampling in the present paper.

In conclusion, the English, French and Dutch versions of the FACIT-F, despite minor DIF, can be reasonably treated as essentially equivalent measures. If our results are replicated, the translations of several items, particularly the Dutch translation of items 7 and 8, should be reconsidered, especially given the influence of the FACIT system in other approaches to measure fatigue in chronic diseases.
